# Building Capacity for the Ethical Conduct of Genetic and Genomic Research in Nigeria: The Vanderbilt-Nigeria Research Ethics Training Program

**DOI:** 10.25259/ijtmrph_95_2024

**Published:** 2025-05-02

**Authors:** Elisa J. Gordon, Zubairu Iliyasu, Elizabeth S. Rose, Fatimah I. Tsiga-Ahmed, Usman J. Wudil, Aishatu L. Adamu, C. William Wester, Muktar H. Aliyu

**Affiliations:** 1 Department of Surgery and Center for Biomedical Ethics and Society, Vanderbilt University Medical Center, Nashville, Tennessee, United States,; 2 Department of Community Medicine, Bayero University/Aminu Kano Teaching Hospital, Kano, Nigeria,; 3 Vanderbilt Institute for Global Health, Vanderbilt University Medical Center, Nashville, Tennessee, United States,; 4 Department of Health Policy, Vanderbilt University Medical Center, Nashville, Tennessee, United States.

**Keywords:** Capacity Building, Global Health, Institutional Review Board, International, Precision Medicine, Research Ethics

## Abstract

**Background and Objective:**

The Vanderbilt-Nigeria Research Ethics Training Program (V-NET) is an innovative research ethics training program that aims to address the increasing demand for research in precision medicine in Africa by building capacity for the ethical design, conduct, and oversight of genetic and genomic research in Nigeria.

**Methods:**

The program includes creation of a Master of Science (MSc) degree program in research ethics, training of 15 MSc students, and the integration of a genomics-focused ethics curriculum into a faculty enrichment program at Vanderbilt University Medical Center (VUMC), benefiting eight Nigerian researchers over 5 years. Twelve Nigerian Institutional Review Board (IRB) members will participate in a practicum at VUMC to enhance their skills in protocol review and administrative procedures. Other capacity-building activities include an annual workshop for IRB members in Nigeria on the protection of human subjects in research, responsible conduct of research, ethics of genetic/genomic research, and review of genomic research protocols (~150 trainees), and the creation of a curricular toolkit in ethics of genetic/genomic research tailored to Nigerian IRB members, in addition to quarterly webinars.

**Results:**

V-NET will equip a group of Nigerian scientists with advanced skills, positioning them as leaders in the ethical design and review of genetic and genomic research within Nigeria’s dynamic research environment.

**Conclusion and Implications for Translation:**

Research ethics training programs are essential for strengthening the ethical design and implementation of clinical and translational research, particularly in low- and middle-income countries.

## INTRODUCTION

Genomic research studies have become increasingly prevalent,^[1–3]^ including in sub-Saharan Africa,^[4,5]^ given advances in technology, increased funding, and growing interest in personalized medicine. Genomic research studies are, however, associated with unique ethical challenges. Ethical dilemmas arising from genomic research include challenges with the informed consent process and protecting confidentiality, return of genetic test results, adequacy of genetic counseling, disclosure of incidental findings, data privacy, future use of stored samples, and protection from harms due to disclosure, among others.^[1,3,6]^ The expanding volume and complexity of genetic and genomic studies call for trained scientists equipped to provide ethics education, guide reviews, and offer expert consultation to safeguard research participants’ rights.

Nigeria, Africa’s most populous nation, is a key contributor to the continent’s scientific research output.^[7–10]^ A study commissioned by the Hastings Center found that 40% of health research ethics committees in Nigeria reported a lack of training in bioethics as a major challenge to adhering to Nigeria’s national code of health research ethics and the World Health Organization’s research ethics guidelines.^[11]^ In addition, the operations and review processes of these committees varied in the extent to which they conformed to these guidelines. Thus, training in ethics governance is urgently needed in Nigeria.

Investments by the Fogarty International Center (FIC) of the U.S. National Institutes of Health (NIH) have contributed to significant growth of research bioethics infrastructure in Nigeria, including the establishment of the National Health Research Ethics Committee (NHREC).^[12]^ There is, however, room for additional work. With a population of approximately 220 million people^[13]^ and at least 250 different ethnic groups, Nigeria is a large and culturally diverse country, with significant regional differences in cultural practices, religion, economic development, genetic diversity, and educational attainment.^[14–16]^ Northern Nigeria is predominantly Islamic and conservative, whereas communities in Southern Nigeria are more westernized, with higher educational attainment and wealth, partly due to earlier exposure to British colonial rule.^[14–17]^ The practice of research ethics is closely related to the local sociocultural norms of host communities. As a result, regional inequalities exist in the quality and extent of research ethics activities, including variability in the degree to which operations of ethics review committees conform to established national and international guidelines.^[11]^

### Objectives

**Figure 1:**
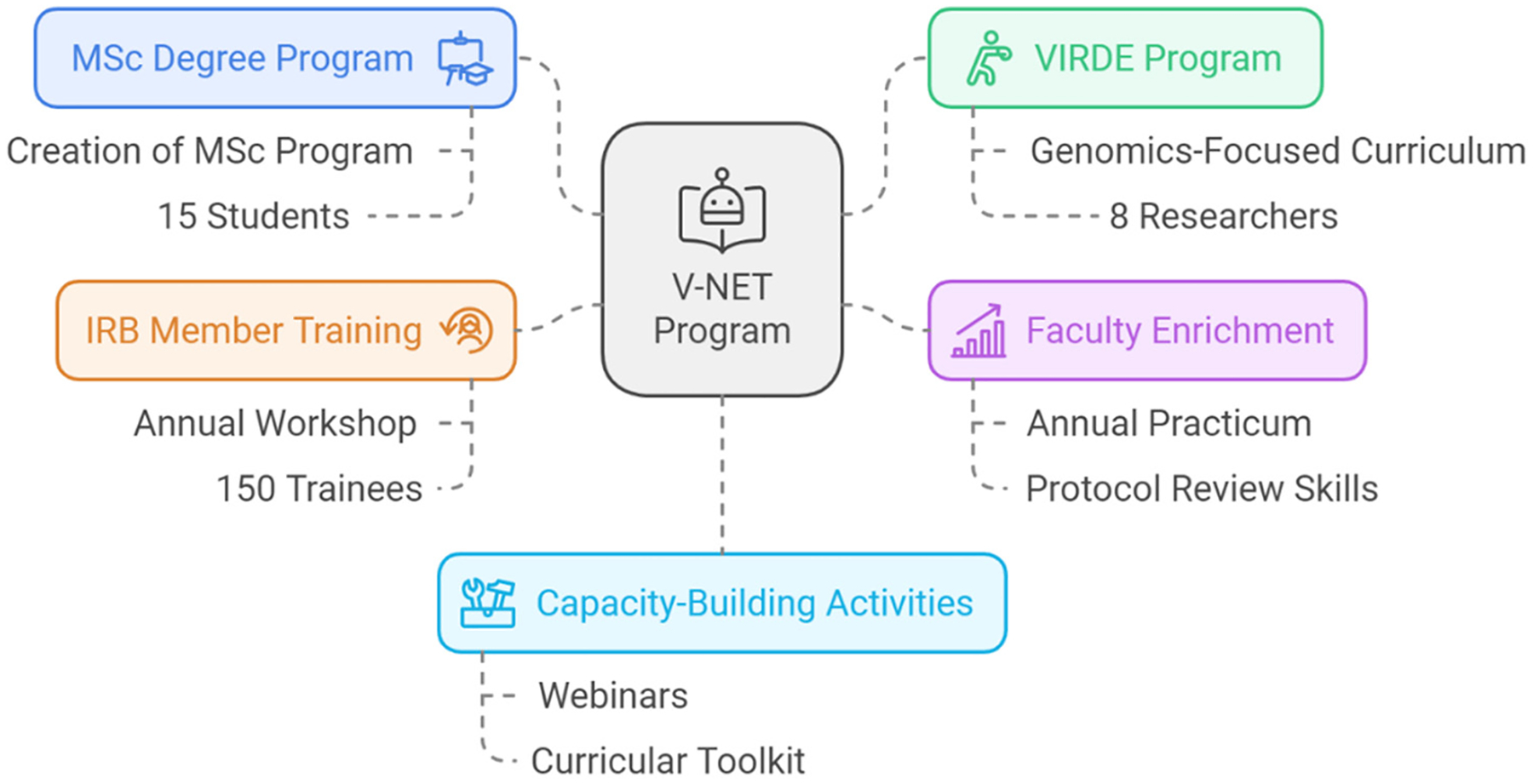
Major components of the Vanderbilt-Nigeria Research Ethics Training (V-NET) program. MSc: Master of Science, VIRDE: Vanderbilt Institute for Research Development and Ethics, IRB: Institutional Review Board.

The goal of the Vanderbilt-Nigeria Research Ethics Training Program (V-NET) is to create a regional hub of expertise in the ethical design, conduct, and oversight of genetic and genomic research in northern Nigeria through interrelated educational, mentoring, and practical training activities [Figure 1]. This paper describes the V-NET implementation protocol, aiming to guide the development of similar ethics training programs in other parts of Africa and enhance ethical standards in genetic and genomic research.

## METHODS

V-NET is a 5-year program that aims to establish the first master’s degree program in research ethics at the Aminu Kano Teaching Hospital (AKTH) and Bayero University Kano (BUK) in northern Nigeria. V-NET will also provide training opportunities in research ethics that include skills development, mentoring, and practicum experiences [Table 1].

### V-NET Administrative Structure

The V-NET administrative structure is organized to ensure effective support and management across the program. The Executive Committee made up of principal investigators and co-investigators, provides strategic leadership. The Training Advisory Committee (TAC), which includes experts in research ethics, advises and supports the program’s initiatives. The Curriculum Development Committee, led by an experienced educator, focuses on developing and refining the curriculum. The Program Coordination Team manages the administrative aspects of V-NET to ensure smooth operations. Faculty mentors from AKTH/BUK assist trainees in selecting, developing, and implementing their research ethics projects, offering guidance and expertise.

### Master’s Degree in Research Ethics

There are currently only two master’s degree programs in research ethics in Nigeria, both established with support from the FIC and located in the southern part of the country. We will establish the first master’s degree program in research ethics in northern Nigeria, home to more than half of Nigeria’s 220 million citizens and a growing scientific research environment.

We performed an initial needs assessment in collaboration with faculty colleagues at BUK.^[18]^ The needs assessment was used to develop competencies that trainees will be expected to gain from the degree program. The assessment helped determine the best format for pedagogy, potential mentors, modalities for incorporation of distance learning modules, and ways to ensure program sustainability.

### Master of Science (MSc) curriculum

The master’s degree curriculum focuses on improving students’ knowledge of the ethical issues associated with the responsible conduct of research (RCR), especially in relation to genetic and genomic research. We emphasize a comprehensive approach to research ethics education that addresses established professional norms in interrelated areas, such as research regulations and oversight regarding human subjects; informed consent in resource-constrained settings; research misconduct; collaborative research, including collaborations with industry; peer review; responsible authorship; conflicts of interest; data acquisition and management; contemporary ethical issues in biomedical research; and the socio-environmental impacts of scientific research, among others.

The MSc students will undertake 30 credit hours of coursework, aligning with the standard requirements for master’s programs at BUK. The curriculum includes didactic instruction, mentorship, a 4-week internship, and a culminating thesis research paper. Program competencies and course objectives will be met through in-class lectures and discussions, interactive group exercises (including case studies), tests, and assignments. Students will be paired with Vanderbilt University Medical Center (VUMC) and AKTH/BUK mentors and, under their guidance, conduct thesis research that results in a quality, publishable paper. In selecting their thesis topic, students will be encouraged to explore ethics topics related to genetic and genomic research.

All curricular materials were written in English and will be evaluated and refined over the 5 years of the grant. The curriculum committee identified and recruited local faculty to teach in the program and develop courses. We will work with BUK to inventory resources and develop a detailed plan to ensure the long-term sustainability of the program beyond the grant period.

### MSc student recruitment

We will enroll 15 students over 4 years, to complete the MSc in Research Ethics at BUK. To be considered for the MSc program position, applicants will be required to have a terminal degree in a health-related or social science field, such as medicine, nursing, biology, sociology, physiology, or psychology. They must also have a minimum grade point average (GPA) of 3.0 and provide evidence of a commitment to a career in academic research. Preference will be given to candidates who are currently employed with AKTH or BUK, as this will allow V-NET to optimize the likelihood of long-term retention of graduates as faculty researchers. Preference will also be given to staff of NHREC, candidates with a demonstrated interest in research ethics, candidates with an academic interest in genetic and genomic research, and individuals who currently work with or have connections to established VUMC-affiliated research programs at AKTH/BUK.

Applicants to the V-NET program will apply through a REDCap^^®^[19]^ online survey form after admission into the MSc program. They will upload their CV, research interests, statement of goals, and letters of recommendation. Top candidates will be invited for online interviews with the V-NET Executive Committee.

Each trainee will have an AKTH/BUK and a VUMC mentor and will meet every 2 weeks with the AKTH/BUK mentor and monthly (virtually) with both mentors. Each student will also have a mentoring committee comprised of the student’s VUMC and AKTH/BUK mentors and the V-NET Training Director, which will meet once every semester. Following each meeting, the student will submit a meeting summary with recommendations that require action from the student.

### Evaluation of the MSc in research ethics

**Figure 2:**
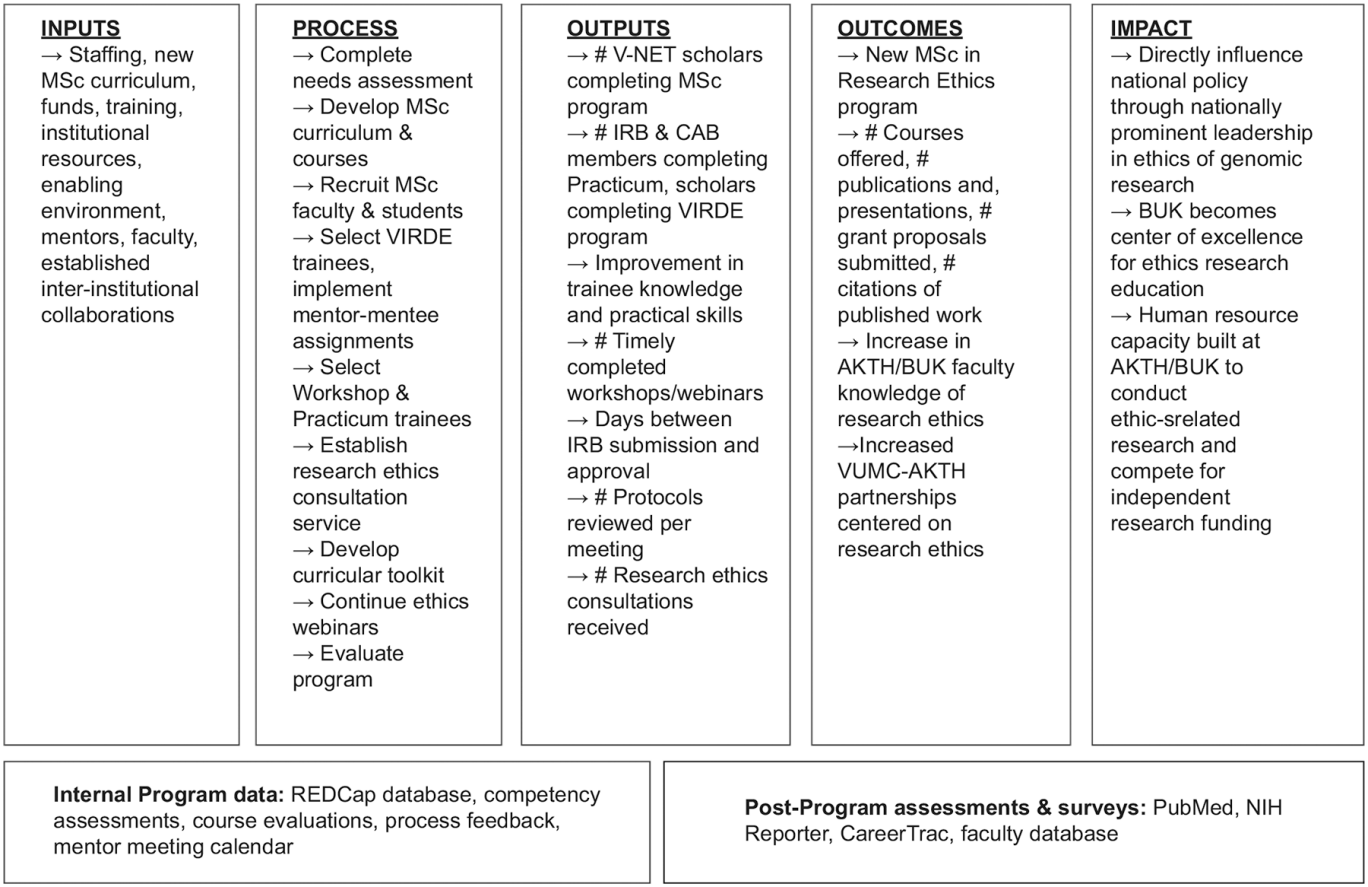
Vanderbilt-Nigeria Research Ethics Training (V-NET) Program: Program Evaluation Plan. MSc: Master of Science, VIRDE: Vanderbilt Institute for Research Development and Ethics, AKTH: Aminu Kano Teaching Hospital, BUK: Bayero University Kano, CAB: Community Advisory Board, IRB: Institutional Review Board.

We will create and distribute course evaluations to students through REDCap^®^ immediately after each course, halfway through the MSc program, and before graduation. We will conduct periodic surveys to gather feedback from course instructors and use the findings to guide quality improvements, as needed. We will also evaluate the MSc program through annual program evaluations. These annual evaluations will include quantitative metrics, such as the number of students and faculty in the program, the student-to-faculty ratio, graduation rates, average time to degree completion, and the number of conference presentations, publications, and citations related to thesis research. We will also track the number of awards received by students and other relevant data. Alongside these quantitative measures, we will gather qualitative data, focusing on student satisfaction and the sense of community and culture within the program. In Year 5, alumni will be surveyed to gather employment data, assess perceptions of the utility of program competencies, and tally the number of scholarly products and research/scientific awards [Figure 2]. All updates will be included in the annual Research Performance Progress Reports and NIH CareerTrac system.^[20]^

We will use Individualized Development Plans (IDPs) to help students articulate career objectives, identify professional development needs, and document their progress. IDPs will be reviewed by the mentoring committees every semester. The IDPs will require completion of all required master’s courses, participation in departmental seminars, V-NET ethics webinars, and relevant workshops. In addition, students must design, propose (defend), and conduct a thesis research project, and present their research at a BUK/AKTH, regional, or other international research conference. Students must also submit at least one first-author manuscript for publication.

Graduates of the MSc degree are expected to enhance ethical standards in research, thereby building public trust and influencing policy development. They will improve the quality and integrity of scientific studies through ethical oversight and interdisciplinary collaboration. These graduates will also be expected to advocate for the rights of vulnerable populations, ensuring that research practices are both innovative and ethically sound.

### Vanderbilt Institute for Research Development and Ethics, “(VIRDE)”

Each October, junior investigators from around the world participate in the 1-month VIRDE faculty enrichment program in Nashville, Tennessee, USA.^[21]^ VIRDE provides intensive training in study design, RCR, survey development, data analysis, data management, and ethical research governance. We will invite 8 Nigerian faculty/researchers or eligible V-NET MSc in Research Ethics alumni to attend VIRDE. VIRDE trainees will complete 12 contact hours of specially tailored coursework in the ethics of genomic research. Trainees will also take part in an intensive grant writing training seminar and one-on-one mentoring, as well as attend VUMC Ethics Center grand rounds and activities. After the VIRDE course, mentors will continue to provide formal one-on-one mentoring.

VIRDE candidates will be AKTH/BUK-affiliated faculty with a demonstrated interest in research ethics (in grant years 1–5) or graduates of the MSc Research Ethics degree at BUK (in grant years 3 and later). We will require candidates to present their draft research proposals through Microsoft Teams. Proposals will be required to have a research ethics focus. Accepted candidates will be paired with a faculty mentor to assist with revising the draft proposal before the participant arrives in Nashville. VIRDE mentors receive a detailed mentoring timeline and are advised on mentoring methods before the course.

### Evaluation of VIRDE training

VIRDE participants will be evaluated by a combination of medium and long-term quantitative and qualitative measures.^[21]^ Quantitative metrics include the number of participants, program completion rates, the number of grants submitted, and the proportion of those grants that receive funding [Figure 2]. Qualitative metrics include participant satisfaction with the courses delivered, mentor feedback, and future plans for networking and collaboration. In addition to routine pre- and post-course evaluations that cover course structure, content, and trainees’ knowledge and skills, we will survey VIRDE participants by email annually to track and assess their career development.

### Practicum at VUMC

Developing skills in ethics protocol review and administration among Institutional Review Board (IRB) staff in low- and middle-income countries is essential for ensuring ethical standards, protecting vulnerable populations, enhancing research quality, and fostering local capacity and international collaborations. Over the course of the grant period, 12 members of the IRBs (AKTH/BUK and NHREC) will take part in a 3-week, in-depth practicum in research ethics at VUMC. Practicum participants will be members of the AKTH and BUK IRBs and NHREC. The primary criteria for participation are membership in the AKTH and BUK IRBs and NHREC, the desire to serve as a research ethics information resource within their institutions and nationally, and the ability to spend 3 consecutive weeks in Nashville to complete the practicum.

The purpose of the practicum is to strengthen the knowledge and skills of Nigerian IRB members in protocol review and administration. Before their arrival in Nashville, participants will be emailed a questionnaire to assess their previous training on ethical dimensions of human subjects’ research. Participants will also be asked to demonstrate their pre-course knowledge of the core concepts and standards of responsible research by taking the standardized multiple-choice Baseline Test of RCR Knowledge,^[22]^ and the online Collaborative Institutional Training Initiative IRB training course modules. We will also encourage participants to explore web-based educational material related to the ethics of genetic and genomic research.^[23,24]^

During the practicum, participants will learn about the structure and organization of the VUMC IRB and will shadow IRB staff members, observing the work of protocol analysts. Participants will also attend the weekly meetings of the VUMC IRB’s committees to observe the committees’ review process and members’ deliberations about protocols submitted by VUMC researchers.

### Practicum evaluation

Evaluations, including practice-based exercises and presentations, will be embedded throughout the practicum. In the case of practical components such as observing protocol reviews, participants will receive immediate and reflective feedback during group discussions. Participants’ learning will be assessed by comparing pre- and post-course scores on the modified Baseline Test of RCR Knowledge.^[22]^ We will also assess each participant’s grasp of the practicum’s objectives by having participants present a 1-hour talk on an issue of their choice in research ethics at the end of the practicum. We will survey the chairs of the AKTH and BUK IRBs and NHREC annually to evaluate the longer-term impact of the practicum on their ethics review operations and processes, including committee conduct, protocol evaluations, and documentation practices.

### Annual Nigeria workshops

We will convene annual, 5-day, intensive, on-site workshops for Nigerian IRB and Community Advisory Board (CAB) members at AKTH in Kano, Nigeria on principles of research ethics, protection of human subjects in research, ethics of genetic and genomic research, and review of research protocols. The course faculty will include practicum graduates, V-NET faculty from VUMC and AKTH/BUK, and other invited experts in human subjects’ research.

The workshops will focus on the historical context of the development of ethical and regulatory standards for biomedical research; the regulations that govern human subjects research and their appropriate application; procedural aspects; and characteristics of protocols that do or do not require review by IRBs. The workshops will also cover practical skills such as how to: respond to review queries from IRBs; assess the risks and benefits of research protocols; determine the comprehensiveness and adequacy of informed consent documents and procedures; produce effective and timely meeting summaries and other records; identify the ethical issues raised in diverse protocols; and propose alternative approaches to addressing or resolving these issues.

Workshop participants will come from a full range of professional and disciplinary backgrounds and will include approximately 30 members and administrators of the AKTH and BUK IRBs, in addition to members of the BUK CAB. We will also reserve a few slots for members of the Kano State Ministry of Health IRB and IRB members from public universities in the region. Participants will register in advance through a secure REDCap^®^ portal and applications will be reviewed by V-NET leadership. Final decisions regarding participation in the workshop will follow similar modalities employed in other training grants.^[25,26]^ To facilitate interaction among participants and faculty, workshops will be limited to a maximum of 30 registrants each, consistent with previous skill-building workshops at AKTH/BUK.^[27–29]^

### Evaluation of workshops

Confirmed registrants will complete a short REDCap^®^ questionnaire to evaluate their previous training on ethical aspects of human subject research. Workshop leaders will use this information to tailor content as needed. Participants will also be asked to complete an online post-workshop survey. The post-workshop survey will include an assessment of course learning objectives, quality of instruction, areas for improvement and unmet needs, contribution to continued interest in research ethics, and post-workshop knowledge of topics. Participants’ pre-workshop knowledge and skills will be compared against their post-workshop levels of knowledge to determine the workshop’s impact and curricular revisions that may be needed.

### Toward sustainability: Toolkit and webinars

Toolkits and webinars are valuable for the sustainability and expansion of ethics training programs because they enhance accessibility, consistency, and engagement. Toolkits provide comprehensive, standardized materials that can be used at the participants’ own pace while webinars offer interactive and cost-effective training that can reach a wide and diverse audience. Both resources support continuous learning, scalability, and the ability to keep training content current and relevant.

We will create, curate, and distribute a curricular toolkit in ethics of genetic and genomic research tailored to Nigerians and other African IRB members and ethics research educators through an online repository. We will revise the tools as needed to ensure they are tailored appropriately to the local context. These repositories will be open to all V-NET participants and be available for wider distribution as desired to promote ongoing professional development of research ethics in the region.

We will also build capacity in research ethics, research integrity, and RCR by delivering 16 quarterly online research ethics webinars conducted by global ethics experts. The quarterly research ethics webinars will last approximately 60 min and will be moderated by V-NET faculty from the U.S. and Nigeria. They will be open to AKTH/BUK IRB and CAB members, MSc in Research Ethics students, and interested members of the public. We will publicize the webinars through our program website and social media. We will make recordings of webinars available to regional ethics committees to foster cross-institutional learning as well as the growth of AKTH/BUK as a leader in research ethics.

### Evaluation of toolkit

We will evaluate the curricular toolkit for its content relevance, accuracy, and usability. Using software analytics embedded in webpages and learning management systems, we will track meta-data regarding page views and downloads of toolkit materials. Online REDCap^®^ feedback forms will be available for users to rate their satisfaction with and utility of materials as well as to provide suggestions for improvement and additional resources. Information will be reviewed annually to ensure the toolkit effectively meets educational goals and user needs, and materials will be updated as needed based on user data and feedback.

### Evaluation of webinars

To ensure that the webinars are informative, engaging, and beneficial for attendees, we will ask participants to complete a brief, anonymous REDCap^®^ survey immediately after each webinar. The survey will include an assessment of learning objectives, content quality, presenter effectiveness, new knowledge gained, overall participant experience, and suggestions for future webinar topics. Feedback from the surveys will be used to determine any changes to the webinar content and process.

### Trainee retention plan

Our in-country selection process will incorporate steps to ensure all V-NET trainees who come to the U.S. for short-term training complete their training and return to Nigeria. We will prioritize staff and faculty with AKTH/BUK appointments and, therefore, secure employment status. In addition, per AKTH and BUK policy, employees approved to proceed on study leave will sign a formal commitment contract to return.

### Dissemination plan

All V-NET training opportunities including workshops will be advertised on our program website, hosted on the VIGH homepage. Dissemination of results will be ongoing throughout the duration of the program, during annual TAC meetings, and appropriate fora, for example, FIC Network meetings. We will publish our experiences from the short-term training/workshops, as we have done with previous training grants.^[27–29]^ Students will be encouraged to disseminate their research findings through peer-reviewed publications and scientific conferences. All published papers will be made available per the NIH public access policy. During the fifth year of the program, larger dissemination events will occur in Nigeria and will emphasize the impact of the program and future sustainability plans.

## RESULTS

In the first year of the grant, we secured final approval from the BUK School of Postgraduate Studies to establish the MSc in Research Ethics program. To inform our curriculum design, we conducted a needs assessment survey and published the findings in a peer-reviewed journal.^[18]^ V-NET Multiple Principal Investigators also attended the NIH Bioethics Network Meeting and Writing Retreat at the NIH/FIC in 2024, where they actively contributed to the West Africa Group’s joint regional paper for a special issue of a major ethics-focused journal. We have delivered three webinars on research ethics topics, with two more scheduled for 2025. Three members of the AKTH IRB committee and NHREC participated in a three-week practicum at the VUMC Center for Biomedical Ethics and Society in October 2024. Furthermore, a five-day research ethics training course, conducted in collaboration with NHREC, was completed in November 2024 to enhance ethical research practices in Nigeria. These initial efforts mark a significant step towards strengthening research ethics education and practice in the region.

## DISCUSSION

We propose a research ethics training program built on a strong and collaborative partnership between U.S. and Nigerian institutions and focused on creating quality learning experiences for trainees. However, we anticipate potential challenges. First, developing a new MSc degree program demands substantial effort and commitment. The team at AKTH/BUK has significant experience in this process and began securing necessary approvals proactively, even before the funding was confirmed. Second, some trainees may express a desire to remain in the U.S. after completing their training and we have noted ways to encourage return to Nigeria to continue strengthening local research capacity. Finally, the academic calendar in Nigeria has historically faced disruptions due to strikes. To mitigate this, we will advocate for flexibility from BUK leadership in designing the MSc in Research Ethics calendar. We will ensure that students have ample time to grasp the material, and we will incorporate distance learning opportunities that are independent of local academic calendars, pending appropriate permissions.

## CONCLUSION AND IMPLICATIONS FOR TRANSLATION

Scientists and regulatory bodies, particularly in low- and middle-income countries, need to understand the ethical implications of the rapidly advancing field of genetics and genomics, as such research will likely expand with technological progress globally. V-NET is an innovative and collaborative training program that will build and maintain the capacity for the ethical design, review, and conduct of genetic and genomic translational research in Nigeria. Lessons learned from this project will help inform the development of similar capacity-building initiatives in related settings.

## Figures and Tables

**Table 1:** Program activities by specific aim and grant year.

Activities	Trainees	Y1	Y2	Y3	Y4	Y5
Aim 1: Create MSc in research ethics program						
Perform needs assessment, develop curriculum, and create a MSc in Research Ethics program at AKTH/BUK		X				
Students matriculate into the 2-year MSc in Research Ethics program	15 students		X	X	X	X
Students conduct mentored thesis projects related to genetic and genomic research (1 year)			X	X	X	X
Aim 2: Develop Short-term Training and Mentoring Opportunities						
4-week Practicum at VUMC for faculty members and select MSc alumni (VIRDE)	8 AKTH/BUK-affiliated faculty and/or MSc alumni	X	X	X	X	X
3-week Practicum for IRB and CAB members at VUMC’s Center for Biomedical Ethics and Society	12 AKTH/BUK IRB and NHREC members	X	X	X	X	X
5-day Annual Research Ethics Course at AKTH/BUK	AKTH/BUK researchers (~150 total)	X	X	X	X	X
Aim 3: Sustain and expand program impact						
Create and distribute genomics research ethics toolkit	Ethicists across SSA		X	X	X	
Host 16 quarterly research ethics webinars	Ethicists and researchers across SSA (~40/webinar)	X	X	X	X	X
Develop and establish a research ethics consultancy service at AKTH/BUK					X	X

AKTH: Aminu kano teaching hospital, BUK: Bayero university kano, CAB: Community advisory board, IRB: Institutional review board, MSc: Master of science, NHREC: National health research ethics committee of Nigeria, SSA: Sub-Saharan Africa, VIRDE: Vanderbilt institute for research development and ethics, VUMC: Vanderbilt university medical center

## Key Messages

1) There is an increasing demand for research in precision medicine in Africa. 2) The practice of research ethics is closely related to the local sociocultural norms of host communities. 3) Training programs in research ethics like the Vanderbilt-Nigeria Research Ethics Training (V-NET) Program will help build capacity in the oversight of genetic and genomic studies.
